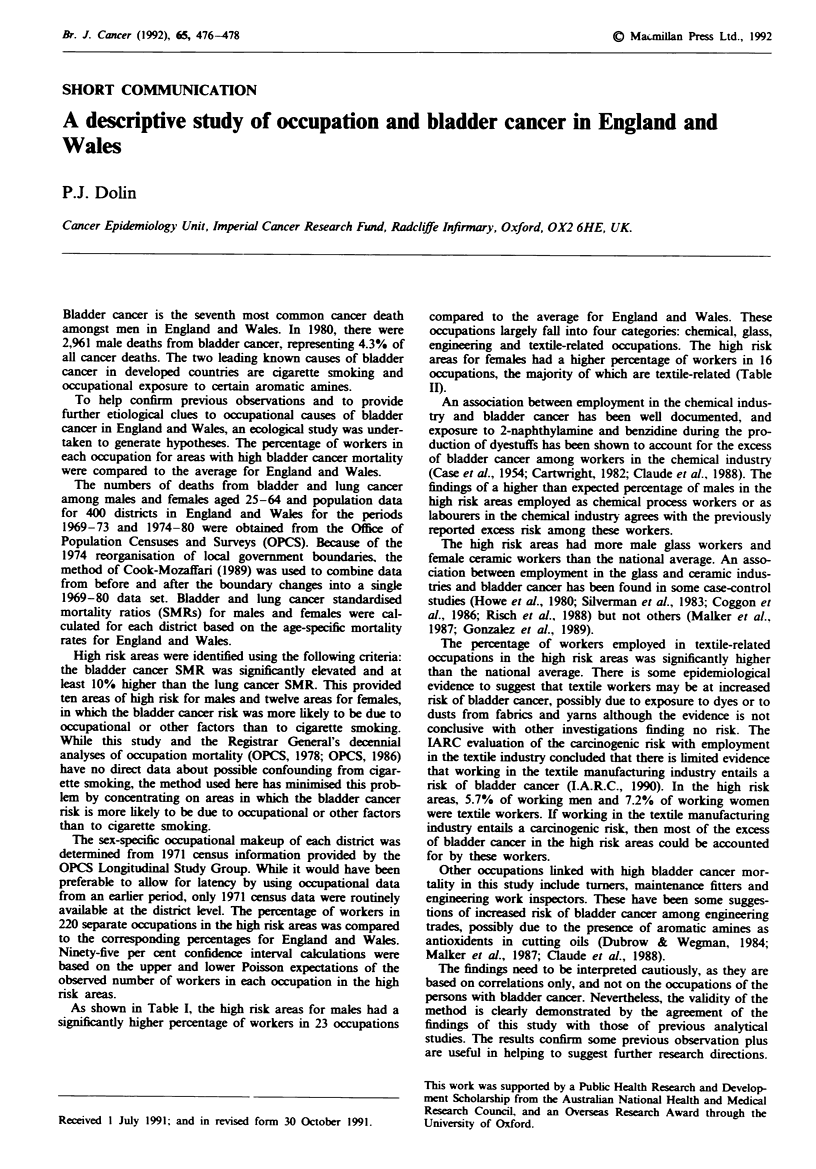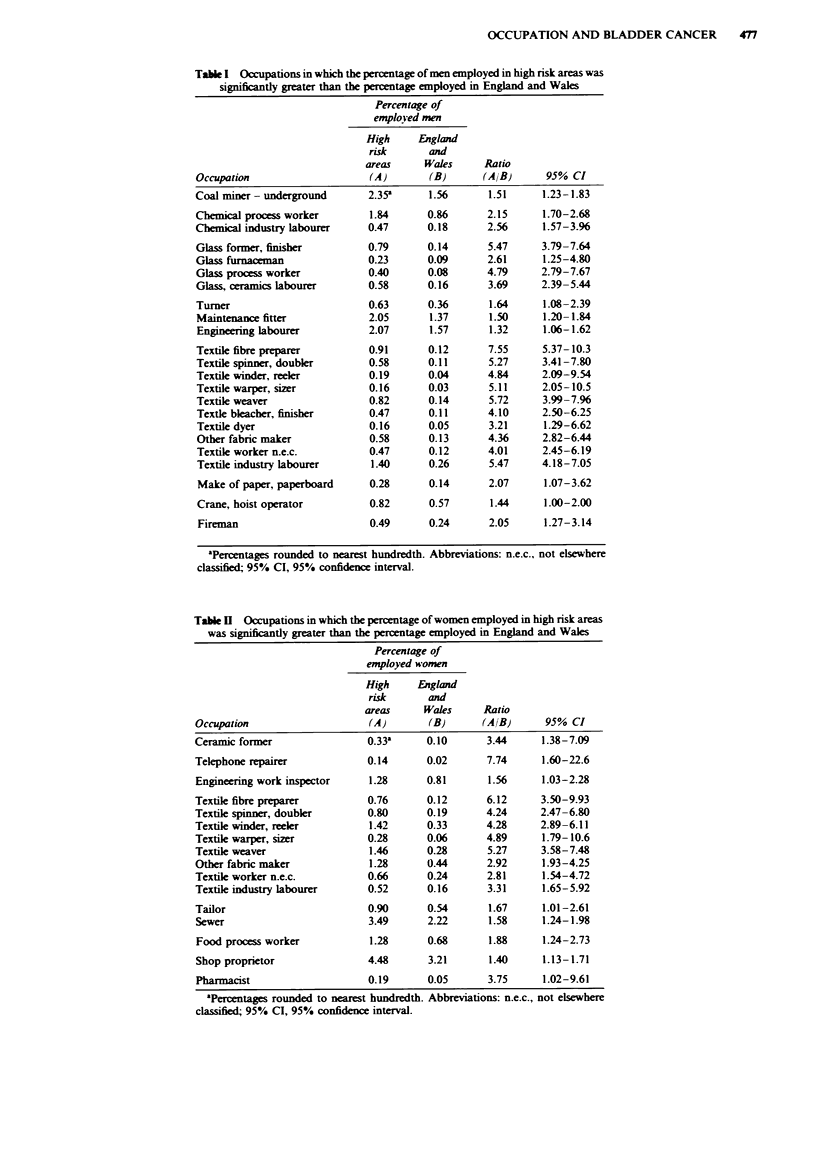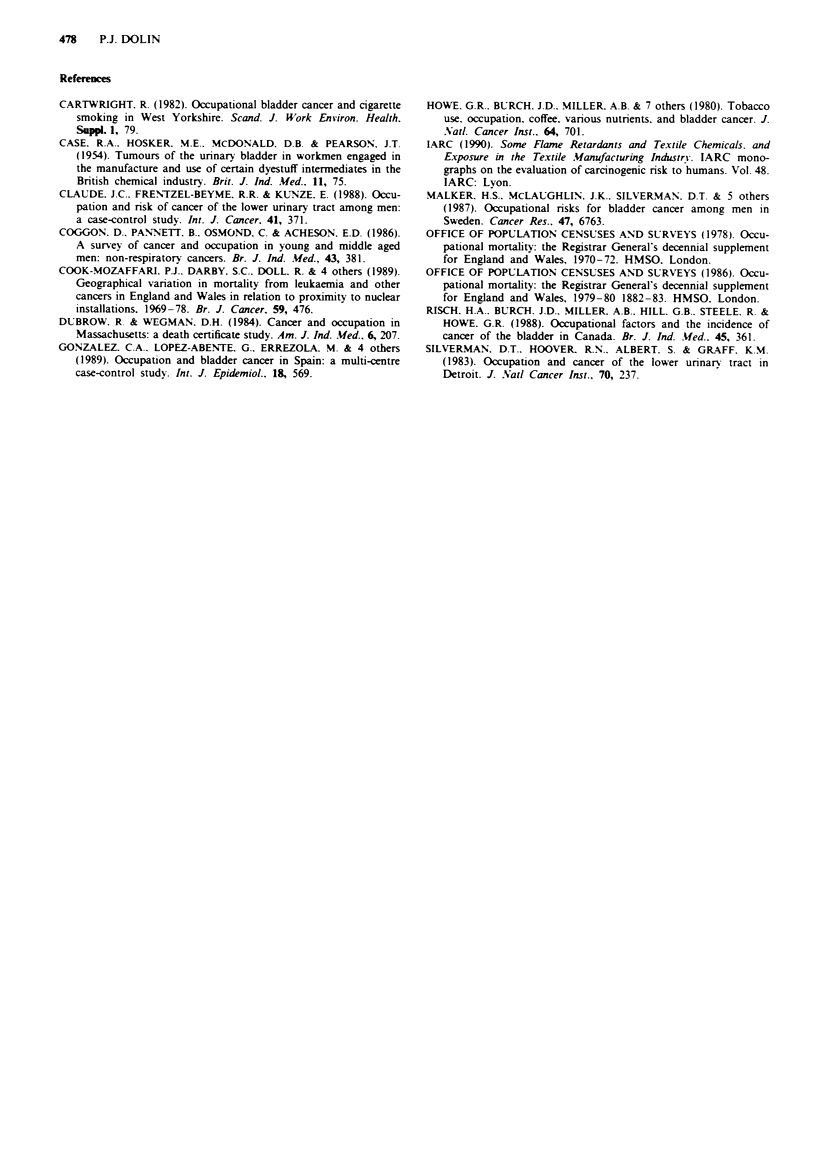# A descriptive study of occupation and bladder cancer in England and Wales.

**DOI:** 10.1038/bjc.1992.96

**Published:** 1992-03

**Authors:** P. J. Dolin

**Affiliations:** Cancer Epidemiology Unit, Imperial Cancer Research Fund, Radcliffe Infirmary, Oxford, UK.


*Br. J. Cancer* (1992), **65**, 476–478 © Macmillan Press Ltd., 1992

SHORT COMMUNICATION

### A descriptive study of occupation and bladder cancer in England and Wales

P.J. Dolin

*Cancer Epidemiology Unit, Imperial Cancer Research Fund, Radcliffe Infirmary, Oxford, OX2 6HE, UK.*

Bladder cancer is the seventh most common cancer death amongst men in England and Wales. In 1980, there were 2,961 male deaths from bladder cancer, representing 4.3% of all cancer deaths. The two leading known causes of bladder cancer in developed countries are cigarette smoking and occupational exposure to certain aromatic amines.

To help confirm previous observations and to provide further etiological clues to occupational causes of bladder cancer in England and Wales, an ecological study was undertaken to generate hypotheses. The percentage of workers in each occupation for areas with high bladder cancer mortality were compared to the average for England and Wales.

The numbers of deaths from bladder and lung cancer among males and females aged 25–64 and population data for 400 districts in England and Wales for the periods 1969–73 and 1974–80 were obtained from the Office of Population Censuses and Surveys (OPCS). Because of the 1974 reorganisation of local government boundaries, the method of Cook-Mozaffari (1989) was used to combine data from before and after the boundary changes into a single 1969–80 data set. Bladder and lung cancer standardised mortality ratios (SMRs) for males and females were calculated for each district based on the age-specific mortality rates for England and Wales.

High risk areas were identified using the following criteria: the bladder cancer SMR was significantly elevated and at least 10% higher than the lung cancer SMR. This provided ten areas of high risk for males and twelve areas for females, in which the bladder cancer risk was more likely to be due to occupational or other factors than to cigarette smoking. While this study and the Registrar General's decennial analyses of occupation mortality (OPCS, 1978; OPCS, 1986) have no direct data about possible confounding from cigarette smoking, the method used here has minimised this problem by concentrating on areas in which the bladder cancer risk is more likely to be due to occupational or other factors than to cigarette smoking.

The sex-specific occupational makeup of each district was determined from 1971 census information provided by the OPCS Longitudinal Study Group. While it would have been preferable to allow for latency by using occupational data from an earlier period, only 1971 census data were routinely available at the district level. The percentage of workers in 220 separate occupations in the high risk areas was compared to the corresponding percentages for England and Wales. Ninety-five per cent confidence interval calculations were based on the upper and lower Poisson expectations of the observed number of workers in each occupation in the high risk areas.

As shown in Table I, the high risk areas for males had a significantly higher percentage of workers in 23 occupations compared to the average for England and Wales. These occupations largely fall into four categories: chemical, glass, engineering and textile-related occupations. The high risk areas for females had a higher percentage of workers in 16 occupations, the majority of which are textile-related (Table II).

An association between employment in the chemical industry and bladder cancer has been well documented, and exposure to 2-naphthylamine and benzidine during the production of dyestuffs has been shown to account for the excess of bladder cancer among workers in the chemical industry (Case *et al.*, 1954; Cartwright, 1982; Claude *et al.*, 1988). The findings of a higher than expected percentage of males in the high risk areas employed as chemical process workers or as labourers in the chemical industry agrees with the previously reported excess risk among these workers.

The high risk areas had more male glass workers and female ceramic workers than the national average. An association between employment in the glass and ceramic industries and bladder cancer has been found in some case-control studies (Howe *et al.*, 1980; Silverman *et al.*, 1983; Coggon *et al.*, 1986; Risch *et al.*, 1988) but not others (Malker *et al.*, 1987; Gonzalez *et al.*, 1989).

The percentage of workers employed in textile-related occupations in the high risk areas was significantly higher than the national average. There is some epidemiological evidence to suggest that textile workers may be at increased risk of bladder cancer, possibly due to exposure to dyes or to dusts from fabrics and yarns although the evidence is not conclusive with other investigations finding no risk. The IARC evaluation of the carcinogenic risk with employment in the textile industry concluded that there is limited evidence that working in the textile manufacturing industry entails a risk of bladder cancer (I.A.R.C., 1990). In the high risk areas, 5.7% of working men and 7.2% of working women were textile workers. If working in the textile manufacturing industry entails a carcinogenic risk, then most of the excess of bladder cancer in the high risk areas could be accounted for by these workers.

Other occupations linked with high bladder cancer mortality in this study include turners, maintenance fitters and engineering work inspectors. These have been some suggestions of increased risk of bladder cancer among engineering trades, possibly due to the presence of aromatic amines as antioxidents in cutting oils (Dubrow & Wegman, 1984; Malker *et al.*, 1987; Claude *et al.*, 1988).

The findings need to be interpreted cautiously, as they are based on correlations only, and not on the occupations of the persons with bladder cancer. Nevertheless, the validity of the method is clearly demonstrated by the agreement of the findings of this study with those of previous analytical studies. The results confirm some previous observation plus are useful in helping to suggest further research directions.

This work was supported by a Public Health Research and Development Scholarship from the Australian National Health and Medical Research Council, and an Overseas Research Award through the University of Oxford.

Received 1 July 1991; and in revised form 30 October 1991.

**Table I** Occupations in which the percentage of men employed in high risk areas was significantly greater than the percentage employed in England and Wales

| | *Percentage of employed men* | | | |
|---|---|---|---|---|
| *Occupation* | *High risk areas (A)* | *England and Wales (B)* | *Ratio (A/B)* | *95% CI* |
| Coal miner – underground | 2.35[a] | 1.56 | 1.51 | 1.23–1.83 |
| Chemical process worker | 1.84 | 0.86 | 2.15 | 1.70–2.68 |
| Chemical industry labourer | 0.47 | 0.18 | 2.56 | 1.57–3.96 |
| Glass former, finisher | 0.79 | 0.14 | 5.47 | 3.79–7.64 |
| Glass furnaceman | 0.23 | 0.09 | 2.61 | 1.25–4.80 |
| Glass process worker | 0.40 | 0.08 | 4.79 | 2.79–7.67 |
| Glass, ceramics labourer | 0.58 | 0.16 | 3.69 | 2.39–5.44 |
| Turner | 0.63 | 0.36 | 1.64 | 1.08–2.39 |
| Maintenance fitter | 2.05 | 1.37 | 1.50 | 1.20–1.84 |
| Engineering labourer | 2.07 | 1.57 | 1.32 | 1.06–1.62 |
| Textile fibre preparer | 0.91 | 0.12 | 7.55 | 5.37–10.3 |
| Textile spinner, doubler | 0.58 | 0.11 | 5.27 | 3.41–7.80 |
| Textile winder, reeler | 0.19 | 0.04 | 4.84 | 2.09–9.54 |
| Textile warper, sizer | 0.16 | 0.03 | 5.11 | 2.05–10.5 |
| Textile weaver | 0.82 | 0.14 | 5.72 | 3.99–7.96 |
| Textle bleacher, finisher | 0.47 | 0.11 | 4.10 | 2.50–6.25 |
| Textile dyer | 0.16 | 0.05 | 3.21 | 1.29–6.62 |
| Other fabric maker | 0.58 | 0.13 | 4.36 | 2.82–6.44 |
| Textile worker n.e.c. | 0.47 | 0.12 | 4.01 | 2.45–6.19 |
| Textile industry labourer | 1.40 | 0.26 | 5.47 | 4.18–7.05 |
| Make of paper, paperboard | 0.28 | 0.14 | 2.07 | 1.07–3.62 |
| Crane, hoist operator | 0.82 | 0.57 | 1.44 | 1.00–2.00 |
| Fireman | 0.49 | 0.24 | 2.05 | 1.27–3.14 |

[a]Percentages rounded to nearest hundredth. Abbreviations: n.e.c., not elsewhere classified; 95% CI, 95% confidence interval.

**Table II** Occupations in which the percentage of women employed in high risk areas was significantly greater than the percentage employed in England and Wales

| | *Percentage of employed women* | | | |
|---|---|---|---|---|
| *Occupation* | *High risk areas (A)* | *England and Wales (B)* | *Ratio (A/B)* | *95% CI* |
| Ceramic former | 0.33[a] | 0.10 | 3.44 | 1.38–7.09 |
| Telephone repairer | 0.14 | 0.02 | 7.74 | 1.60–22.6 |
| Engineering work inspector | 1.28 | 0.81 | 1.56 | 1.03–2.28 |
| Textile fibre preparer | 0.76 | 0.12 | 6.12 | 3.50–9.93 |
| Textile spinner, doubler | 0.80 | 0.19 | 4.24 | 2.47–6.80 |
| Textile winder, reeler | 1.42 | 0.33 | 4.28 | 2.89–6.11 |
| Textile warper, sizer | 0.28 | 0.06 | 4.89 | 1.79–10.6 |
| Textile weaver | 1.46 | 0.28 | 5.27 | 3.58–7.48 |
| Other fabric maker | 1.28 | 0.44 | 2.92 | 1.93–4.25 |
| Textile worker n.e.c. | 0.66 | 0.24 | 2.81 | 1.54–4.72 |
| Textile industry labourer | 0.52 | 0.16 | 3.31 | 1.65–5.92 |
| Tailor | 0.90 | 0.54 | 1.67 | 1.01–2.61 |
| Sewer | 3.49 | 2.22 | 1.58 | 1.24–1.98 |
| Food process worker | 1.28 | 0.68 | 1.88 | 1.24–2.73 |
| Shop proprietor | 4.48 | 3.21 | 1.40 | 1.13–1.71 |
| Pharmacist | 0.19 | 0.05 | 3.75 | 1.02–9.61 |

[a]Percentages rounded to nearest hundredth. Abbreviations: n.e.c., not elsewhere classified; 95% CI, 95% confidence interval.

### References

CARTWRIGHT, R. (1982). Occupational bladder cancer and cigarette smoking in West Yorkshire. *Scand. J. Work Environ. Health*, **Suppl. 1**, 79.

CASE, R.A., HOSKER, M.E., MCDONALD, D.B. & PEARSON, J.T. (1954). Tumours of the urinary bladder in workmen engaged in the manufacture and use of certain dyestuff intermediates in the British chemical industry. *Brit. J. Ind. Med.*, **11**, 75.

CLAUDE, J.C., FRENTZEL-BEYME, R.R. & KUNZE, E. (1988). Occupation and risk of cancer of the lower urinary tract among men: a case-control study. *Int. J. Cancer*, **41**, 371.

COGGON, D., PANNETT, B., OSMOND, C. & ACHESON, E.D. (1986). A survey of cancer and occupation in young and middle aged men: non-respiratory cancers. *Br. J. Ind. Med.*, **43**, 381.

COOK-MOZAFFARI, P.J., DARBY, S.C., DOLL, R. & 4 others (1989). Geographical variation in mortality from leukaemia and other cancers in England and Wales in relation to proximity to nuclear installations, 1969–78. *Br. J. Cancer*, **59**, 476.

DUBROW, R. & WEGMAN, D.H. (1984). Cancer and occupation in Massachusetts: a death certificate study. *Am. J. Ind. Med.*, **6**, 207.

GONZALEZ, C.A., LOPEZ-ABENTE, G., ERREZOLA, M. & 4 others (1989). Occupation and bladder cancer in Spain: a multi-centre case-control study. *Int. J. Epidemiol.*, **18**, 569.

HOWE, G.R., BURCH, J.D., MILLER, A.B. & 7 others (1980). Tobacco use, occupation, coffee, various nutrients, and bladder cancer. *J. Natl. Cancer Inst.*, **64**, 701.

IARC (1990). *Some Flame Retardants and Textile Chemicals, and Exposure in the Textile Manufacturing Industry*. IARC monographs on the evaluation of carcinogenic risk to humans. Vol. 48. IARC: Lyon.

MALKER, H.S., MCLAUGHLIN, J.K., SILVERMAN, D.T. & 5 others (1987). Occupational risks for bladder cancer among men in Sweden. *Cancer Res.*, **47**, 6763.

OFFICE OF POPULATION CENSUSES AND SURVEYS (1978). Occupational mortality: the Registrar General's decennial supplement for England and Wales, 1970–72. HMSO, London.

OFFICE OF POPULATION CENSUSES AND SURVEYS (1986). Occupational mortality: the Registrar General's decennial supplement for England and Wales, 1979–80 1882–83. HMSO, London.

RISCH, H.A., BURCH, J.D., MILLER, A.B., HILL, G.B., STEELE, R. & HOWE, G.R. (1988). Occupational factors and the incidence of cancer of the bladder in Canada. *Br. J. Ind. Med.*, **45**, 361.

SILVERMAN, D.T., HOOVER, R.N., ALBERT, S. & GRAFF, K.M. (1983). Occupation and cancer of the lower urinary tract in Detroit. *J. Natl Cancer Inst.*, **70**, 237.